# Supplementary figures and images for: Whole exome-seq and RNA-seq data reveal unique neoantigen profiles in Kenyan breast cancer patients
Source: Front Oncol. 2024 Dec 11;14:1444327. doi: 10.3389/fonc.2024.1444327 (PMC11668681; doi:10.3389/fonc.2024.1444327)

**A**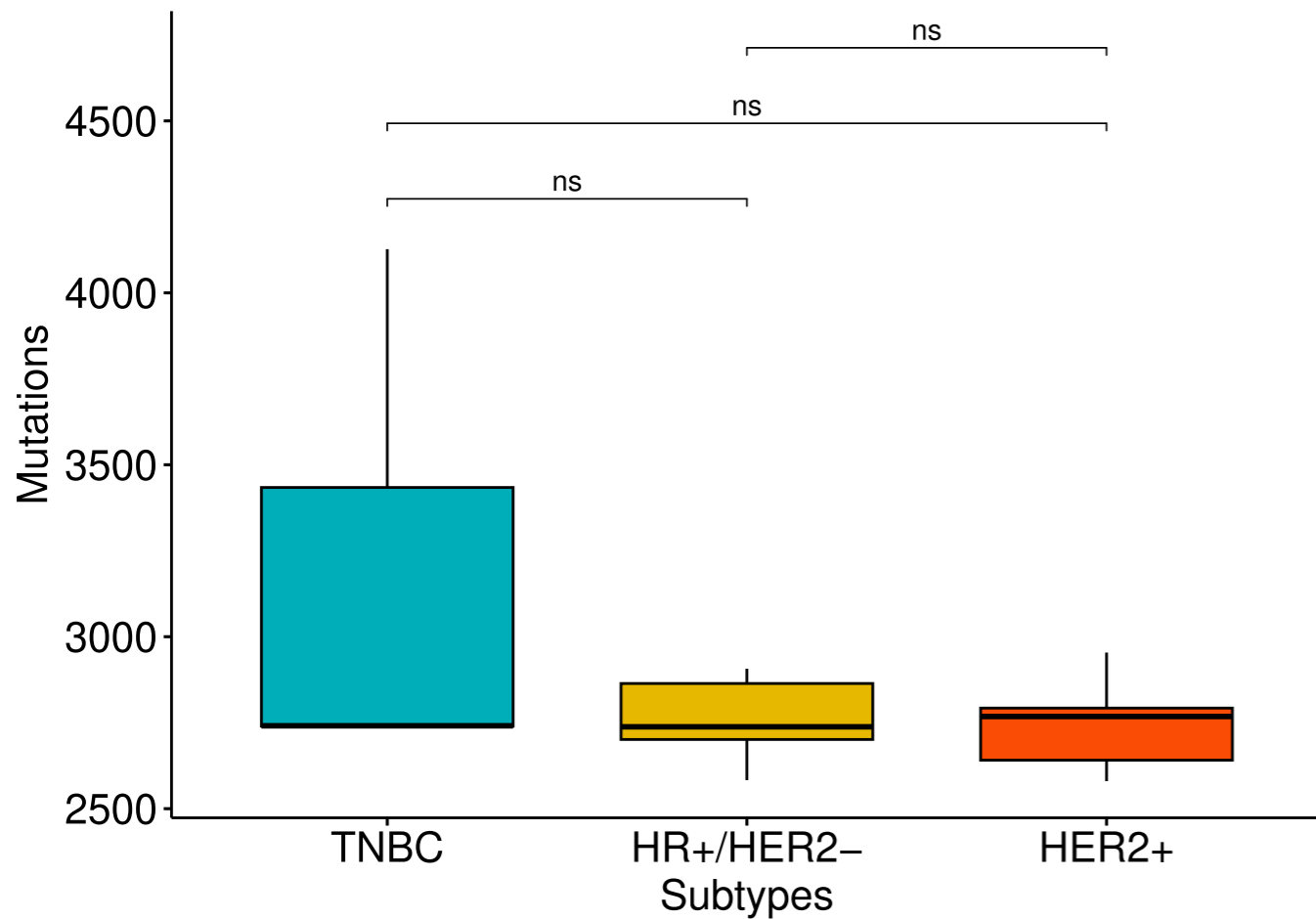**B**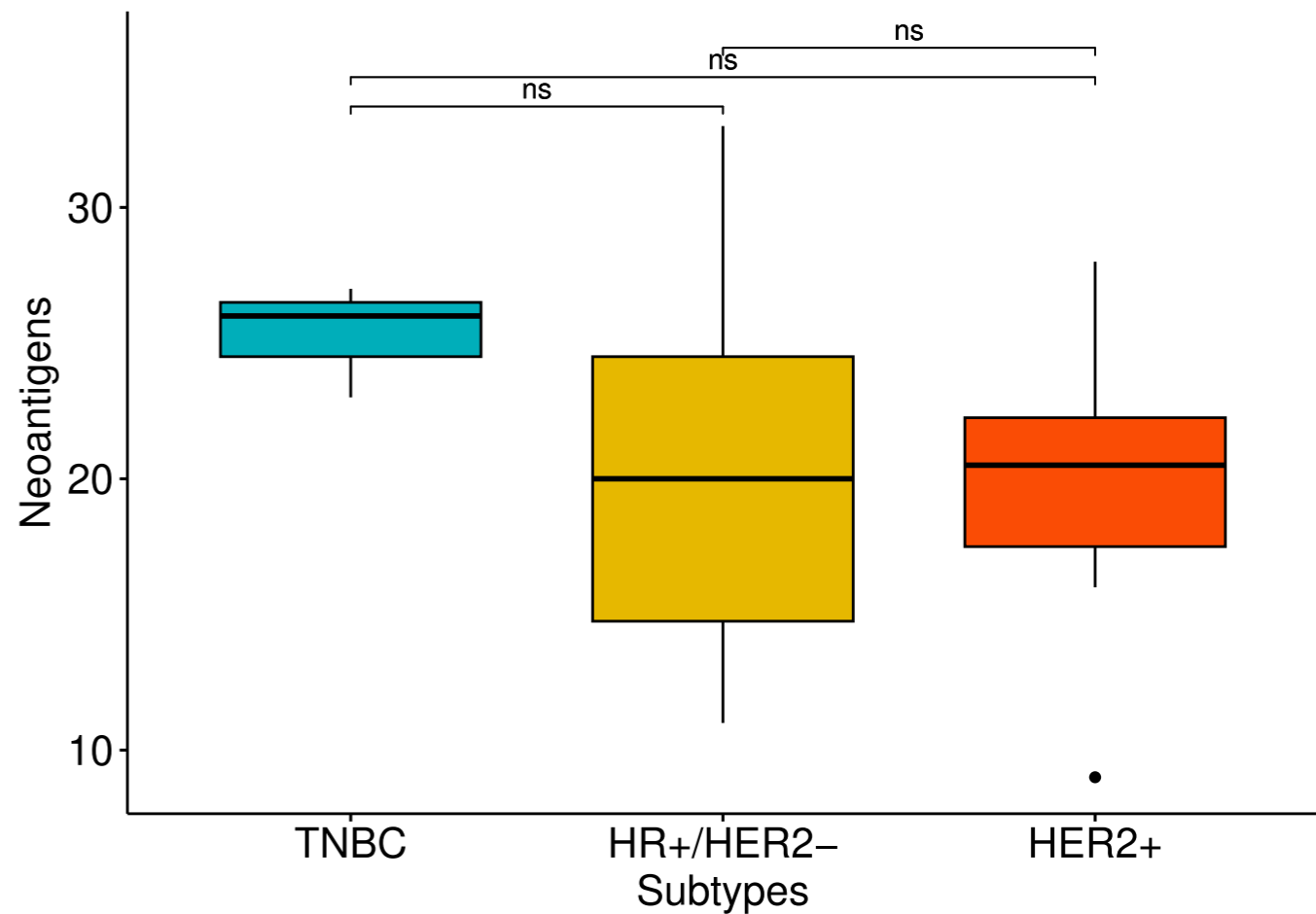

Supplement: Supplementary Figure 1 — Statistical pairwise test (Wilcoxon’s test) for differences in mutational burden (A) and neoantigens counts (B) for the 23 samples. [file Image1.pdf]
